# Revisiting of the essential steps of the mini-culotte technique

**DOI:** 10.1093/ehjcr/ytaf623

**Published:** 2025-11-29

**Authors:** Ezgi Gültekin Güner, Koray Çiloğlu, Ahmet Güner

**Affiliations:** Department of Cardiology, Istanbul Mehmet Akif Ersoy Thoracic and Cardiovascular Surgery Training and Research Hospital, Turgut Özal Bulvari No: 11, 34303, Kucukcekmece, Istanbul, Turkey; Department of Cardiology, Istanbul Mehmet Akif Ersoy Thoracic and Cardiovascular Surgery Training and Research Hospital, Turgut Özal Bulvari No: 11, 34303, Kucukcekmece, Istanbul, Turkey; Department of Cardiology, Istanbul Mehmet Akif Ersoy Thoracic and Cardiovascular Surgery Training and Research Hospital, Turgut Özal Bulvari No: 11, 34303, Kucukcekmece, Istanbul, Turkey

To the editor,

We have read with great interest entitled ‘Case report: Pierced-balloon technique for navigating difficult side branch angulation in a critical left main coronary artery bifurcation disease’ by Nanjappa V, *et al*.^1^ We commend our colleagues for their diligent efforts to develop an innovative strategy and the treatment approach to provide the best possible care for this complex left main (LM) bifurcation disease. However, we believe that several key points need to warrant further discussion.

First of all, the European Bifurcation Club (EBC) recommends an algorithmic approach for baseline side branch wiring in challenging anatomies. In cases where standard wires are unsuccessful owing to severe stenoses and unfavourable angulation (such as this case), the following steps should be followed: (i) pullback technique; (ii) pre-shape the tip of the wire according to the bifurcation anatomy. The curves typically used for this purpose are: (a) a single bend with a short (2–3 mm) tip, (b) a single bend with a long (4–6 mm) tip, (c) a single wide-angled bend, and (d) a double bend shape; (iii) use of hydrophilic and more torquable guidewires (such as Samurai or Sion Black); (iv) the hairpin technique; and (v) the use of microcatheters, especially dual-lumen microcatheters; and (vi) plaque modification with balloon or rotablation as a last resort (*Figure 1*).^2^ We understand that the authors encountered equipment issues; however, we believe that the use of a hydrophilic, high-torque guidewire with a hairpin technique can significantly increase the success rate of successful wiring in such challenging anatomies. A step-by-step approach by interventionalists to wiring issues in challenging anatomies may reduce potential complications (*Figure 1*).

**Figure 1:**
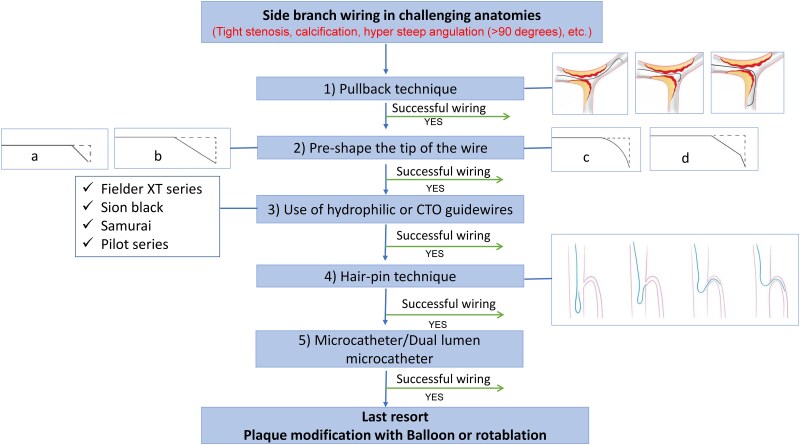
The summary of side-branch wiring techniques for challenging anatomies. CTO, chronic total occlusion.

Second, we once again congratulate the operators on their creative approach. However, we believe that interventional cardiologists performing this technique may run the risk of losing the side-branch wire's position during removal of the main vessel balloon (punctured balloon) from the guide catheter. This is particularly likely if a hydrophilic wire is positioned in the side-branch.

Third, when we revisit the mini-culotte technique, we note that: (i) pre-dilatation, particularly at the LM bifurcation, should be performed with a 1:1 size non-compliant balloon; (ii) after side-branch stenting, proximal optimization technique should be performed with a non-compliant balloon appropriate for the proximal size of the main vessel.^3^ If intravascular imaging is unavailable, the POT balloon diameter should be determined according to Finet's law.^3^ Furthermore, if the culotte technique is to be used, the technical characteristics of the stents should be well known, because it should be kept in mind that the napkin-ring formation can lead to fatal consequences.^4,5^

In summary, in patients with complex LM bifurcation lesions, the algorithmic approach of the EBC to wiring challenging anatomy may improve operators’ side-branch wiring success. Furthermore, adequate implementation of the basic steps in 2-stent bifurcation techniques plays a crucial role in ensuring optimal clinical outcomes.

## Data Availability

No new data were generated or analysed in support of this research.
